# AQuA Tools: clear and reliable BEDPE operations for 3D genomics

**DOI:** 10.1093/bioinformatics/btaf510

**Published:** 2025-09-17

**Authors:** Maharshi Chakraborty, Laura Morgan, Quin Scacheri, Alexandra D’Yan, Hyunmin Kim, Yaw Asante, Diana Chin, Olivia Corradin, Peter Scacheri, Richard Sallari, Berkley Gryder

**Affiliations:** Axiotl Inc., Cleveland, OH 44106, United States; Axiotl Inc., Cleveland, OH 44106, United States; Axiotl Inc., Cleveland, OH 44106, United States; Axiotl Inc., Cleveland, OH 44106, United States; Department of Genetics and Genome Sciences, Case Western Reserve University, Cleveland, OH, 44106, United States; Department of Genetics and Genome Sciences, Case Western Reserve University, Cleveland, OH, 44106, United States; Department of Genetics and Genome Sciences, Case Western Reserve University, Cleveland, OH, 44106, United States; Whitehead Institute of Biomedical Research, Cambridge, MA 02142, United States; Massachusetts Institute of Technology, Cambridge, MA 02139, United States; Department of Genetics and Genome Sciences, Case Western Reserve University, Cleveland, OH, 44106, United States; Axiotl Inc., Cleveland, OH 44106, United States; Department of Genetics and Genome Sciences, Case Western Reserve University, Cleveland, OH, 44106, United States

## Abstract

**Motivation:**

The genome interacts with itself within the volume of the cell nucleus to process information. These interactions mediate signal integration, gene regulation, and cell identity. The identification of new therapeutic targets from non-coding disease-associated variants relies critically on correctly assigning variants to genes through 3D interactions. Experimental techniques in 3D genomics, such as HiC and HiChIP, allow the mapping of interactions through sequencing. Bioinformatics for 3D genomics contends primarily with contact matrices that contain interaction frequencies for all possible element pairs, and BEDPE files that store element pairs that interact. Whereas the tools available for processing linear genomic data are mature, operating on contact matrices and BEDPE files remains cumbersome, opaque, and error-prone, as researchers have had to shoehorn tools originally designed for linear data. A genome arithmetic designed from the ground up for 3D genomics does not yet exist.

**Results:**

We present AQuA Tools, a suite of shell- and R-based command-line tools that provide a set of core operations on contact matrices and BEDPE files motivated by key questions in population genetics, cancer research, and precision medicine. We have designed our core operations to be clear, reliable, intuitive and versatile. Core operations can be chained together along with standard UNIX commands. Our goal is to make AQuA Tools easy for the novice to learn and the go-to choice for power users. We hope our tools will motivate more researchers to use 3D genomic data in their projects.

**Availability and implementation:**

We provide and maintain AQuA Tools at https://github.com/axiotl/aqua-tools.

## 1 Introduction

### 1.1 Landscape of 3D genomics

Understanding disease mechanisms relies critically on how the genome interacts with itself. In variant-to-function dissection of non-coding disease-associated variants identified in genome-wide association studies (GWAS), assigning the right gene to a risk variant can make the difference between a new therapeutic target and a dead-end research project. In cancer research, physical interactions reveal the targets of mutations, rewiring of core regulatory circuits and deregulation of genes in highly rearranged tumors. Assignments of coding variants to genes are assumed to be cell-type invariant. If a variant overlaps an exon it is assigned to the gene in all cell types. Unfortunately this is not the case for non-coding variants, where gene assignments vary by cell type. A variant in an enhancer will only be assigned to a gene if the enhancer is active and looped to the gene promoter in a given cell type. Consequently, proper burden tests for germline non-coding variants will require complete maps of 3D interactions for all cell types in the human body. In all of these cases, mapping interactions in the right context is becoming increasingly recognized as a priority, with both comprehensive references and richly diverse 3D genomic datasets emerging as a key bottleneck in the elucidation of disease mechanisms.

**Figure 1.**
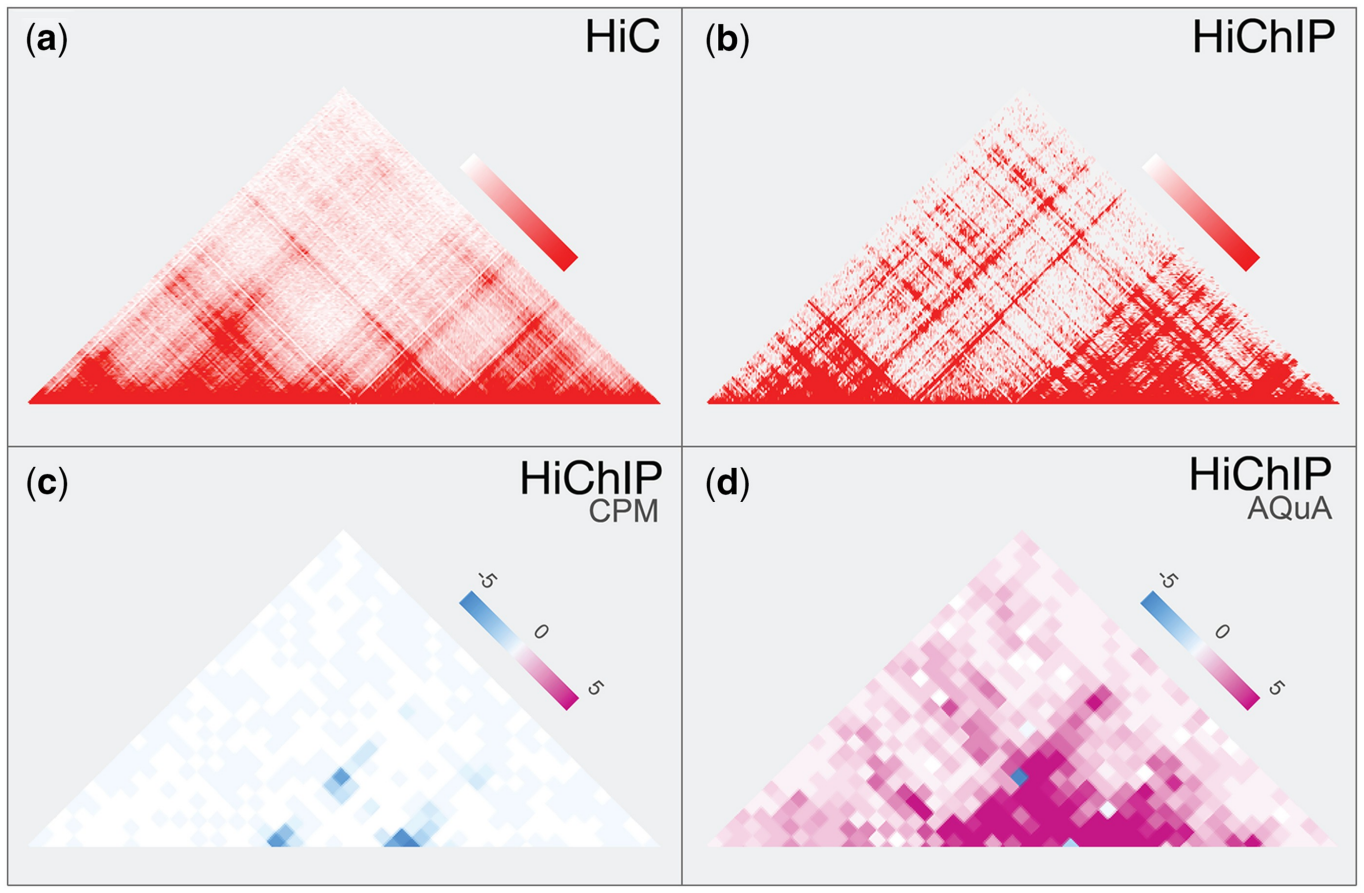
(a) Contact plot showing interactions between genomic loci in chr2:64–65 Mb in GM12878 cell-line using HiC and (b) interactions in the same cell-line and region using H3K27ac HiChIP. The selective enrichment for interactions bound by a factor of interest in HiChIP results in more visible 3D structure. (c) Direct comparison (subtraction) between contract matrices of DMSO and entinostat treated RH4 cells in chr11:17.6–17.8 Mb, normalized using the CPM metric showing overall loss of contacts for H3K27ac HiChIP, (d) however when reference normalized using spike-in mouse genome reveals a global increase of contacts surrounding a single lost loop.

Experimental techniques in 3D genomics, notably HiC and HiChIP, have enabled the generation of high-resolution chromatin interactions maps up to the resolution of 1 kb (Rao *et al.* 2014, Mumbach *et al.* 2016). HiC maps all interactions genome-wide (Fig. 1a). HiChIP adds a chromatin immunoprecipitation step which selectively enriches interactions mediated by specific proteins or histone marks. If the precipitation target is against histones with acetylated tails (H3K27ac), the resulting contact matrix reveals genome-wide interactions between elements with either enhancer or promoter-like signatures (Fig. 1b). Where HiC contact matrices look like mountain ranges of solid triangles representing interacting regions, HiChIP contact matrices exhibit a plaid pattern of criss-crossed flares, which are long, narrow projections of high frequency contacts emanating from a few elements with many interactions.

An important advancement to HiChIP, and the namesake for our tools, is the Absolute Quantification of chromatin Architecture (AQuA) HiChIP protocol (Gryder *et al.* 2020). The standard normalization for HiC and HiChIP is counts per million (CPM), where interaction read counts are divided by the total number of reads and then scaled by a million. As HiChIP selectively captures interactions mediated by a protein or histone mark, global differences in the total amount of the pulled-down molecule between samples can confound the comparison. AQuA-HiChIP uses an orthogonal spike-in genome like mouse, to enable absolute quantitative insights with regards to the molecule selected for immunoprecipitation. For example, in the study of cells treated with drugs that alter histone acetylation globally, such as HDAC inhibitors, AQuA can flip how we interpret the gain or loss of contacts between enhancers and their target genes (Fig. 1c and d).

### 1.2 Bioinformatics for 3D genomics

After library generation and read-pair sequencing, the pipeline for obtaining a contact matrix (.hic or .cool) has been highly standardized using frameworks such as Juicer, HiC-Pro and the nf-core consortium (Servant *et al.* 2015, Durand *et al.* 2016, Ewels *et al.* 2020). These pipelines perform the heavy lifting operations such as alignment of read pairs to a reference genome, filtering invalid fragments, generating quality control reports and binning reads into a matrix. More recently, pairtools has provided flexible command-line utilities for processing sequencing data from HiC experiments, offering modular tools that can be chained together for customizable preprocessing pipelines (Open2C *et al.* 2024). Visualization tools such as Juicebox enable fast loading and interactive exploration of high-resolution, gigabyte-scale contact matrices (Robinson *et al.* 2018). These foundational processing steps are shared between HiC and HiChIP. However, for most labs the complexity of 3D genomic analysis lies in the steps between generating contact matrices and visualizing loci of interest. It is between these two steps that the hypothesis-driven analysis is performed. The analysis that will yield the publishable biological insight will be a sequence of operations on BED and BEDPE files to query, filter, transform and summarize the values stored in one, two or a cohort of contact matrices.

### 1.3 A need for BEDPE operations

A parallel between linear genomics and 3D genomics can be drawn by the issues addressed by BEDTools (Quinlan and Hall 2010). Linear signal tracks produced from experiments like ChIP-seq and ATAC-seq have a mature suite of tools that enable researchers to tinker with the resulting files from their experiments and ask novel biological questions. BEDTools enables intuitive operations on BED files which now have become a common language of genome arithmetic such as intersect, merge, etc.

An equivalent set of operations for genome arithmetic on BEDPE files still does not exist. The following question is easy to formulate in English: “Filter all interactions found in a set of samples based on the presence of a transcription factor binding site in one interacting element and a gene promoter in the other, then rank filtered interactions based on the ratio in contact frequency between treatment and control samples”. However, translating the question into code still requires a substantial degree of 3D genomics and bioinformatics expertise. Conversely, after translation the original question will not be readily apparent from the code.

Existing tools such as FAN-C and HiCExplorer support format conversion, matrix summarization and region plotting (Kruse *et al.* 2020, Wolff *et al.* 2020). While comprehensive, they do not yet offer a set of operations as foundational as BEDTools. AQuA tools are our proposal for an equivalent arithmetic that is native to 3D genomics, with contact matrices and BEDPE files as the central data structures. Our core operations create, extract, query, transform, summarize or visualize BEDPE files using contact matrices, BED files or other BEDPE files as inputs.

## 2 Features and methods

### 2.1 Operations that create a BEDPE

AQuA tools provide two methods for creating BEDPE files: *build* and *extract*. These tools are often the first step in a 3D genomic analysis. *Build* is used when linear elements of interest are known in advance, and we are interested in evaluating their interactions based on a contact matrix. *Extract* is used to obtain a BEDPE directly from a contact matrix.

#### 2.1.1 build_bedpe (BEDs → BEDPE)

**Figure 2.**
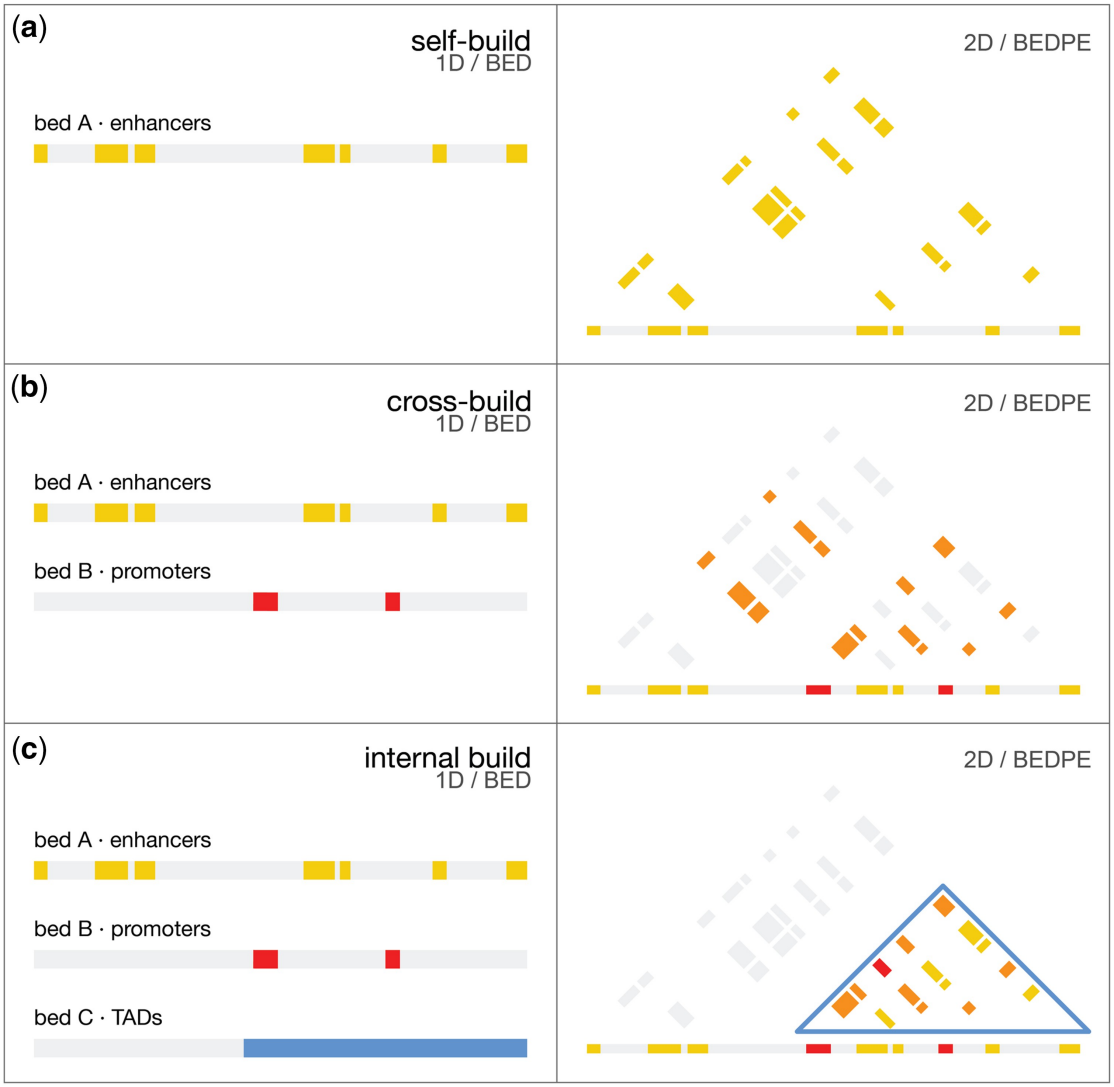
(a) The *build_bedpe* tool allows users to move from 1D to 2D genomic space. The simplest transformation is to “pop out” all pairwise combinations of elements from a single 1D BED file to a 2D BEDPE file. (b) Frequently we are interested in pairwise interactions between elements with distinct characteristics, in this example enhancers and promoters. Cross building using *build_bedpe* between gene TSSs and regulatory elements is the most common first step in 3D genomic analyses. (c) Computing all pairwise interactions between elements genome-wide can quickly overwhelm downstream analyses. Restricting the build operation by distance or by using a third BED file specifying the intervals in which to build focuses resources on biologically relevant interactions and lightens the computational load.

Using linear elements known in advance, *build_bedpe* constructs element pairs between all regions contained in two BED files, outputting a BEDPE (Fig. 2a and b). Pairings can be constrained using a third BED file, typically representing TADs, or by specifying minimum and maximum distances between pairs (Fig. 2c). This makes a canvas of paired elements that can then be used to query a contact matrix.

#### 2.1.2 extract_bedpe (matrix → BEDPE)

**Figure 3.**
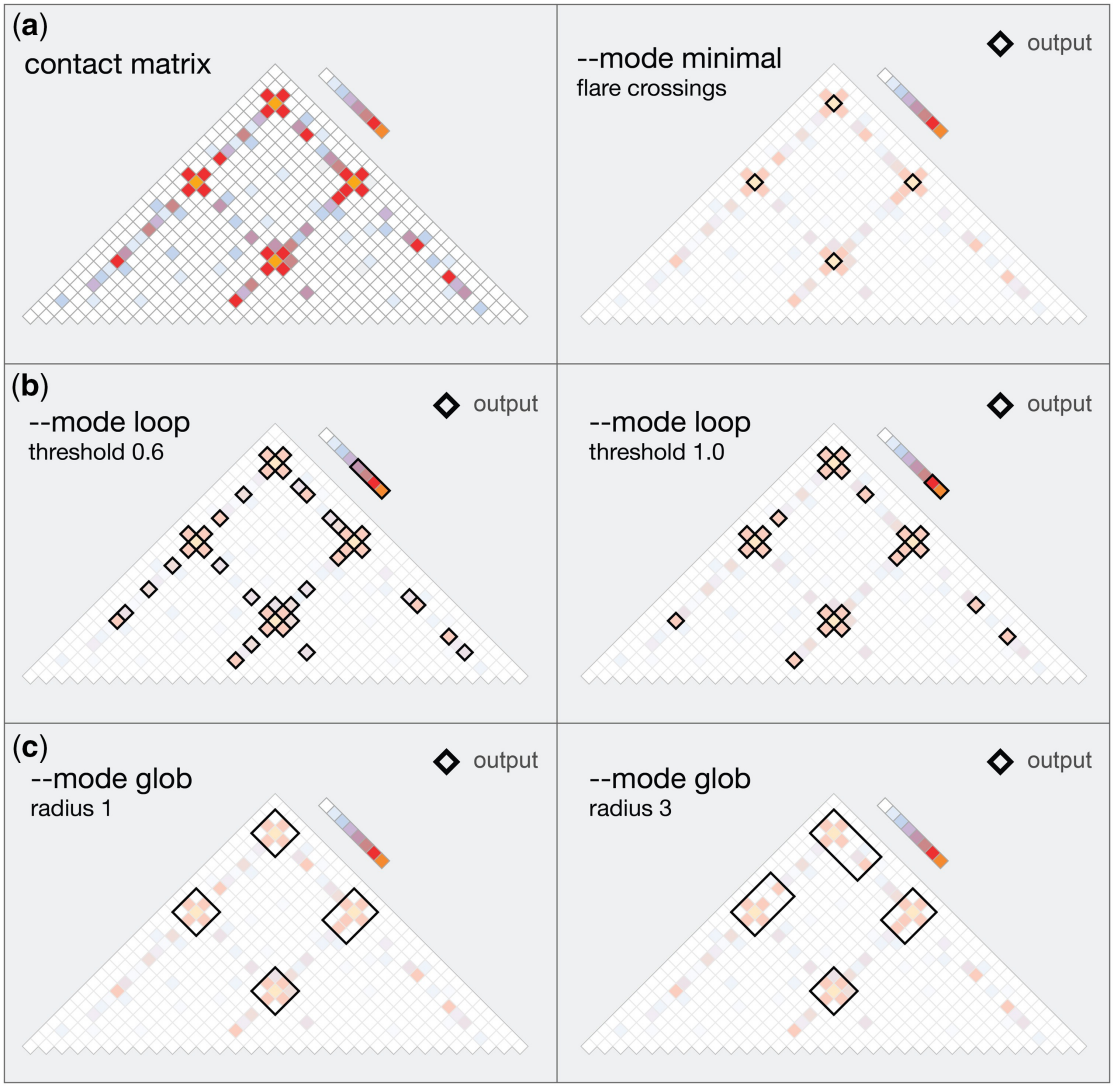
Against a contact matrix, *extract_bedpe* takes a range or TAD file as input and identifies 3D structures by transforming each bin value, irrespective of its distance to the diagonal, into a 0–1 numeric space. This mitigates the distance-dependent interaction decay typical of HiC/HiChIP and allows for single value thresholding and binarization of bins across the matrix. extract_bedpe can report (a) flare crossings, which are contact bins where long projections of HiChIP signal that emanate from the diagonal cross paths. (b) single bin loops, entries in the matrix that exceed a user supplied threshold (c) globbed single bin loops, that agglomerates individual bins that exceed the threshold into larger BEDPE structure. The radius parameter determines the maximum number bins between conjoined structures above the threshold.

Without using linear elements known in advance, *extract_bedpe* identifies interacting element pairs from a .hic file and a user-defined score threshold. Scores are calculated using inherent normalization (Supplementary Material, available as supplementary data at *Bioinformatics* online), which creates a uniform numeric space using sample-specific power-law distributions. Inherent normalization rescales each matrix bin value (1 and 5 kb resolutions) into a 0–1 range, independent of genomic distance, with zero denoting the standard contact frequency between non-interacting active elements and one denoting the standard contact frequency between interacting active regulatory elements. A uniform numeric space therefore allows for meaningful extraction of contact bins, which the tool can report as granular resolution-sized bins, agglomeration of bins in the matrix, or “flare crossings,” which are bins that intersect at long and narrow projections of contact signal often seen in HiChIP matrices (Fig. 3).

### 2.2 Operations that transform a BEDPE

The operations *query*, *union*, *cluster* and *intersect* take a BEDPE file and either output summary metrics, change coordinates, or attach new columns that indicate interaction, membership or intersection.

#### 2.2.1 query_bedpe (BEDPE → BEDPE)

**Figure 4.**
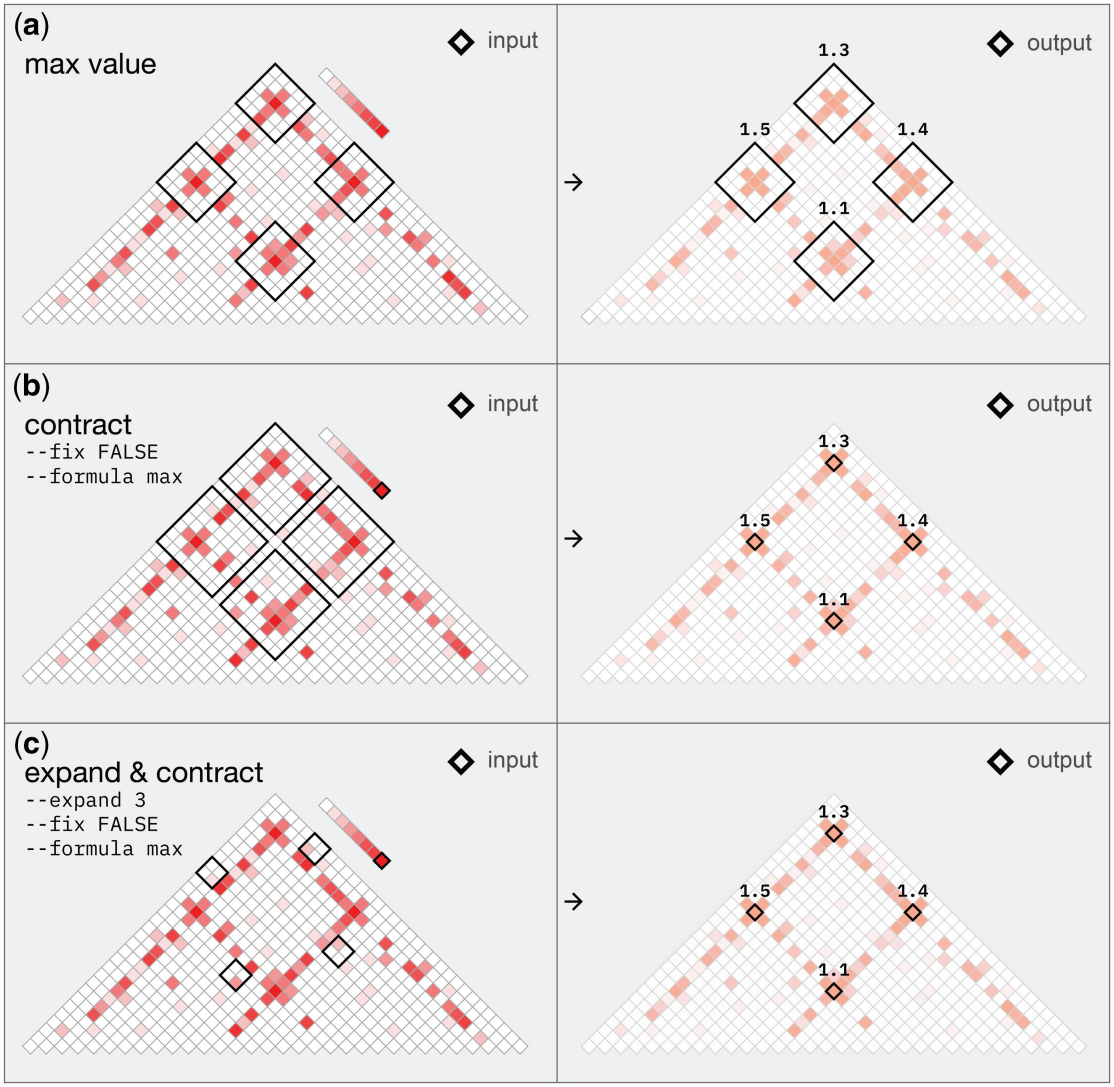
*query_bedpe* takes a contact matrix and a BEDPE as inputs. Using default parameters it appends an additional column to the BEDPE with the numerical values found in the contact matrix. (a). In this example it returns the maximum value within each BEDPE entry, which is the single bin contact value with the highest read count. (b) Through parameter—formula, the tool can provide other numeric values for each BEDPE entry, such as the contact value of the most central bin, sum of all contact values or the average. The tool can also modify the BEDPE entries based on the values in the contact matrix. Parameter—fix FALSE will return BEDPE entries corresponding to the supplied formula (max or center). (c) Similarly, the tool can search for max values by expanding the size of the input BEDPE entries in bin units.

After creating a BEDPE file, *query_bedpe* returns contact matrix values in CPM or AQuA numeric spaces, using four arithmetic methods (center, maximum, sum, or mean). A second .hic file can be included to calculate delta values between samples, for case and control or treated and untreated study designs. Contact values are appended to the input BEDPE and returned as output, with input coordinates either remaining fixed or updating based on the chosen arithmetic formula (Fig. 4).

#### 2.2.2 intersect_bedpe (BEDPE → BEDPE)

**Figure 5.**
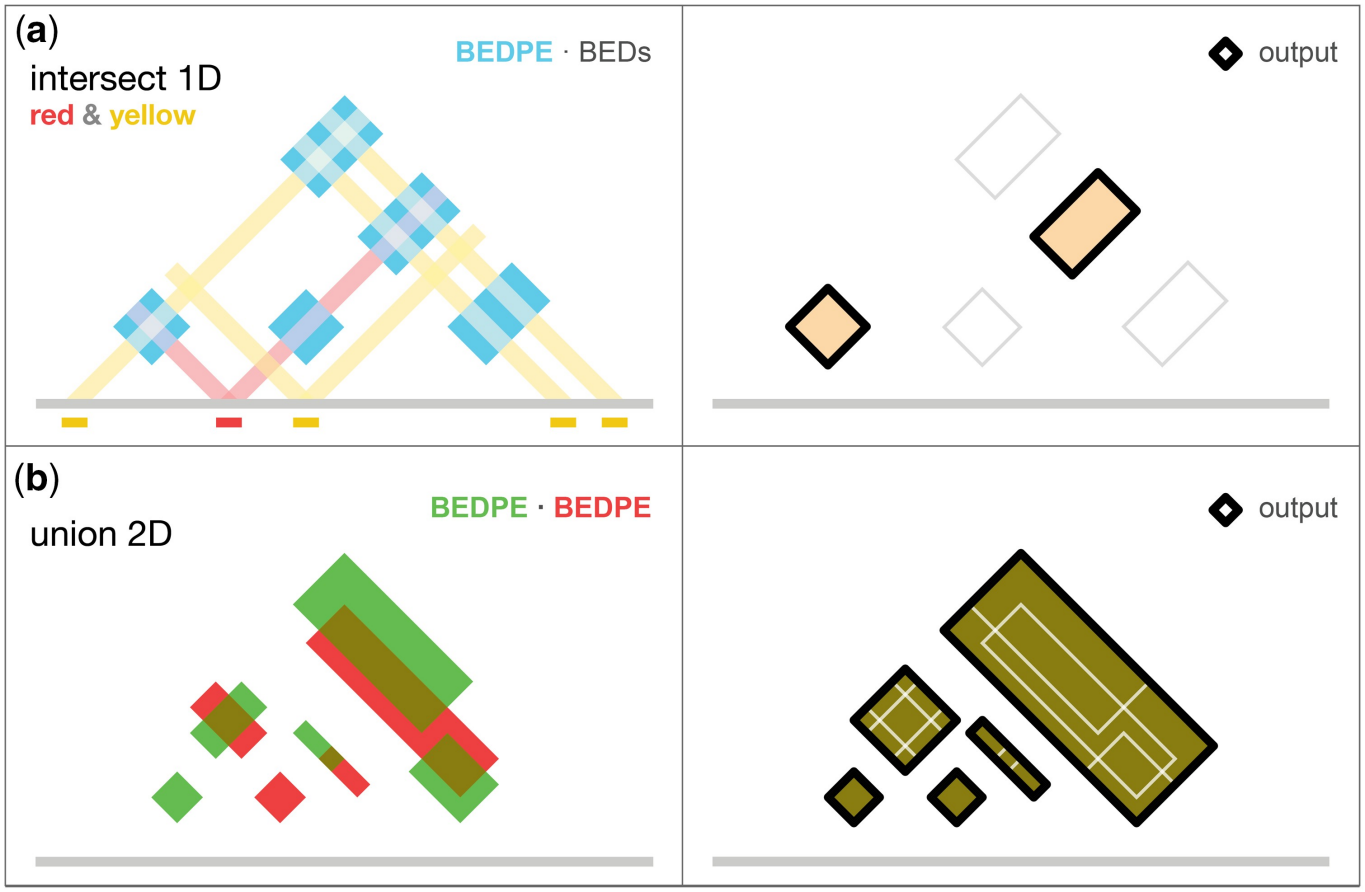
AQuA tools provide the basic operations of intersection and union in one and two dimensions. (a) A BEDPE file in 2D (blue) is intersected with two BED files in 1D (red and yellow). (b) Two BEDPE files are processed to obtain the union of all regions. The genomic union of overlapping coordinates spanning multiple rows and files into a single row in the output BEDPE creates areas that are greater than the geometric union.

*intersect_bedpe* identifies and classifies interactions in a BEDPE file that intersect with regions in a BED file, allowing researchers to focus on interactions involving specific genomic features (Fig. 5a). Rows can be classified based on BED intersections with the upstream, downstream, or both elements of a BEDPE. For example, if P is a BED file containing promoters and E is a BED file containing enhancers, a BEDPE row classified as P-E tells the researcher that the upstream foot intersects with a promoter and the downstream foot intersects with an enhancer. Simple command-line tools like grep can allow researchers to quickly filter the output for enhancer-promoter loops designated as P-E or E-P intersection types. Additional features for *intersect_bedpe* include boolean reporting, absence detection, and the option to output intersecting BED rows instead of BEDPE rows.

#### 2.2.3 union_bedpe (BEDPEs → BEDPE)

Given two or more BEDPE files, *union_bedpe* merges BEDPE coordinates by identifying and resolving overlapping interactions and outputting the union of a set of interactions (Fig. 5b). This is intended for integrating data from various experiments or replicates. For example, using *union_bedpe* on the output of two or more calls of *extract_bedpe* creates a common scaffold in BEDPE format that contains the 2D union of all loops supplied, which can then be used to query contact values using *query_bedpe*.

#### 2.2.4 cluster_bedpe (BEDPE → BEDPE)

**Figure 6.**
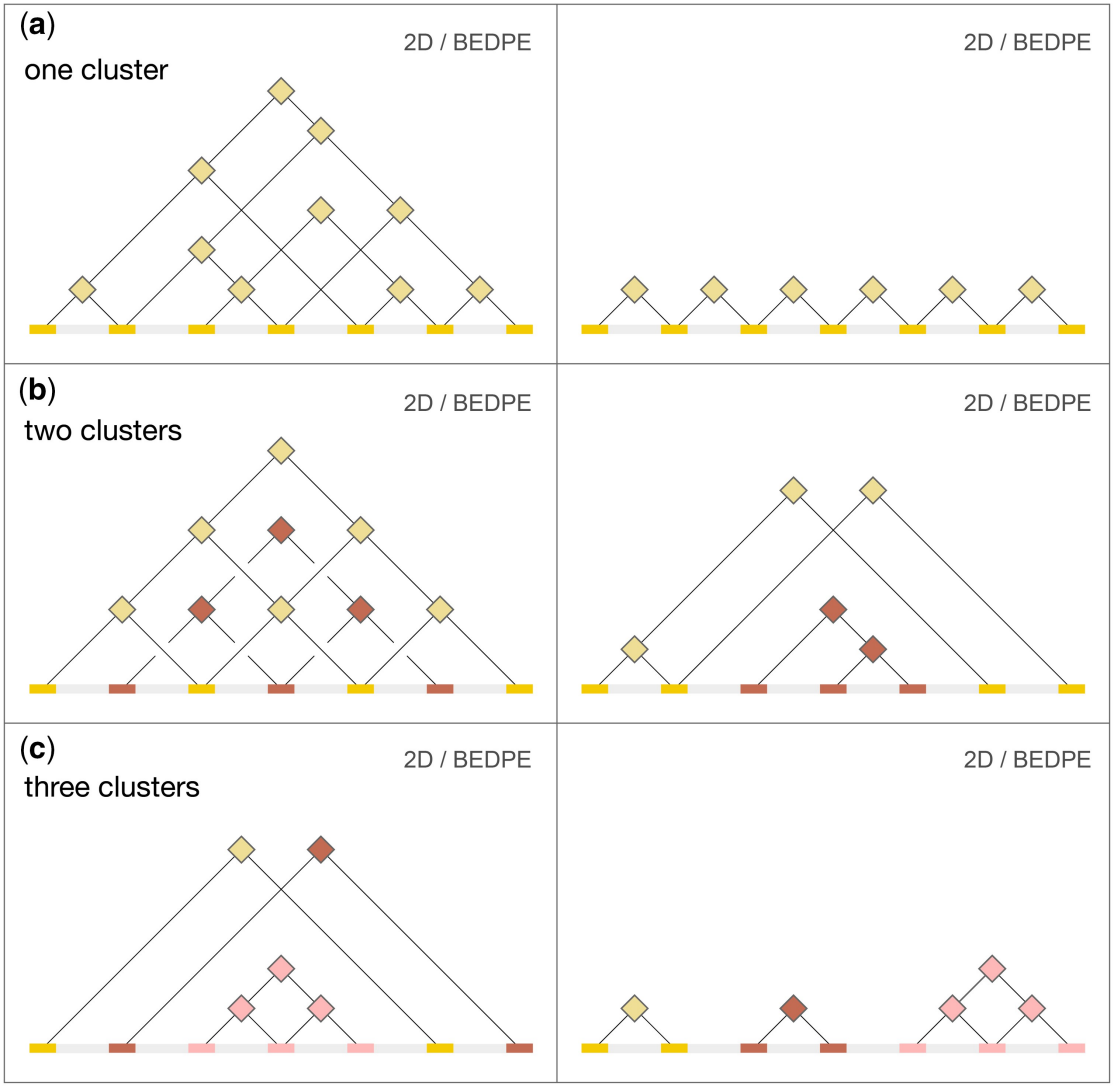
The *cluster_bedpe* tool identifies clusters of interconnected 1D elements based on their 2D pairwise interactions. (a) Clusters of elements can be fully connected, with every possible pairwise interaction showing evidence of a chromatin loop, partially connected, with any subset of pairwise elements supported by loops (left), and minimally connected or “daisy-chained” (right). (b) Clusters are defined purely on the graph structure of nodes (1D elements) and edges (2D loops). Two clusters can be functionally disjoint and yet interleaved across the 1D genome (left). Similarly, the range of one cluster can be fully contained within the range of another (right). (c) We define a pairwise interaction between two elements as our minimal cluster. HiChIP data contains a broad range of cluster complexity. However, the majority of clusters tend to present as with non-overlapping ranges (right).

Within a locus, elements can be connected by loops in a variety of ways, ranging from a sparsely connected daisy-chain to a fully connected network of highly overlapping BEDPE rows. These structures indicate a set of elements that coalesce in 3D space. Using graph theory language, we can refer to genomic elements as nodes and to chromatin loops as edges. Nodes and edges come together to form a small graph or network. More formally, among all the nodes and edges present in the HiChIP sample genome-wide, we are interested in calling connected components or subgraphs. In the context of this paper we refer to these structures using the general term “clusters.” Given a BEDPE file, *cluster_bedpe* assigns membership tags as a 7th column to the BEDPE, based on shared overlap of BEDPE coordinates (Fig. 6). Creating clusters from overlapping interactions provides a flexible, context-specific view of genome organization, capturing dynamic regulatory relationships that TADs may miss.

### 2.3 Operations that summarize a BEDPE

#### 2.3.1 annotate_cluster (BEDPE → BED)

*annotate_cluster* takes a 7-column BEDPE output of *cluster_bedpe* and produces a table where each row provides a summary of each cluster’s properties and intersections with reference genome annotations. For each cluster in the input BEDPE, the output cluster properties include: (i) cluster coordinates, (ii) number of participating loops, (iii) total CPM contact frequency, (iv) total range of cluster, (v) total size cluster loops, (vi) number of FANTOM TSSs, and (vii) number of ENCODE 3 candidate cis-regulatory elements (cCREs).

### 2.4 Operations that visualize a BEDPE

Visualization tools in AQuA Tools expand upon basic visualization operations developed by the Juicer team (Durand *et al.* 2016), but with added normalization compatibility and many additional parameters for increased customization.

#### 2.4.1 plot_contacts

*plot_contacts* is a tool used to visualize the upper triangle of a contact matrix for a given range in PDF format. At minimum, *plot_contacts* requires a sample name, a genomic range, and a genome build. Alternative to specifying a genomic range, a gene name can be provided and the tool will automatically center the plot around the gene. *plot_contacts* can create interchromosomal plots if either an interchromosomal range or two genes on different chromosomes are provided. Two-sample delta plots visually compare the differences in chromatin interactions between two samples. Additional optional parameters allow for extensive customization, including resolution adjustments, numeric matrix output, annotation overlays, BEDPE highlights, and control over color scale.

#### 2.4.2 plot_virtual_4C

*plot_virtual_4C* operates very similarly to *plot_contacts*, and for a given genomic range plots the upper triangle of the contact matrix and the virtual 4C profile of a supplied coordinate of interest.

#### 2.4.3 plot_APA

*plot_APA* is a wrapper for Juicer’s aggregate peak analysis (APA) tool with additional customizable parameters, normalization methods, and delta comparison plot capabilities. The tool centers and aligns regions supplied from a BEDPE file and piles-up the sub-matrices, effectively “skewering” all loops. By default, *plot_apa* outputs an APA plot in PDF format, a histogram of pair distances from the diagonal, and the numerical matrix used to create the APA plot.

## 3 Example

**Figure 7.**
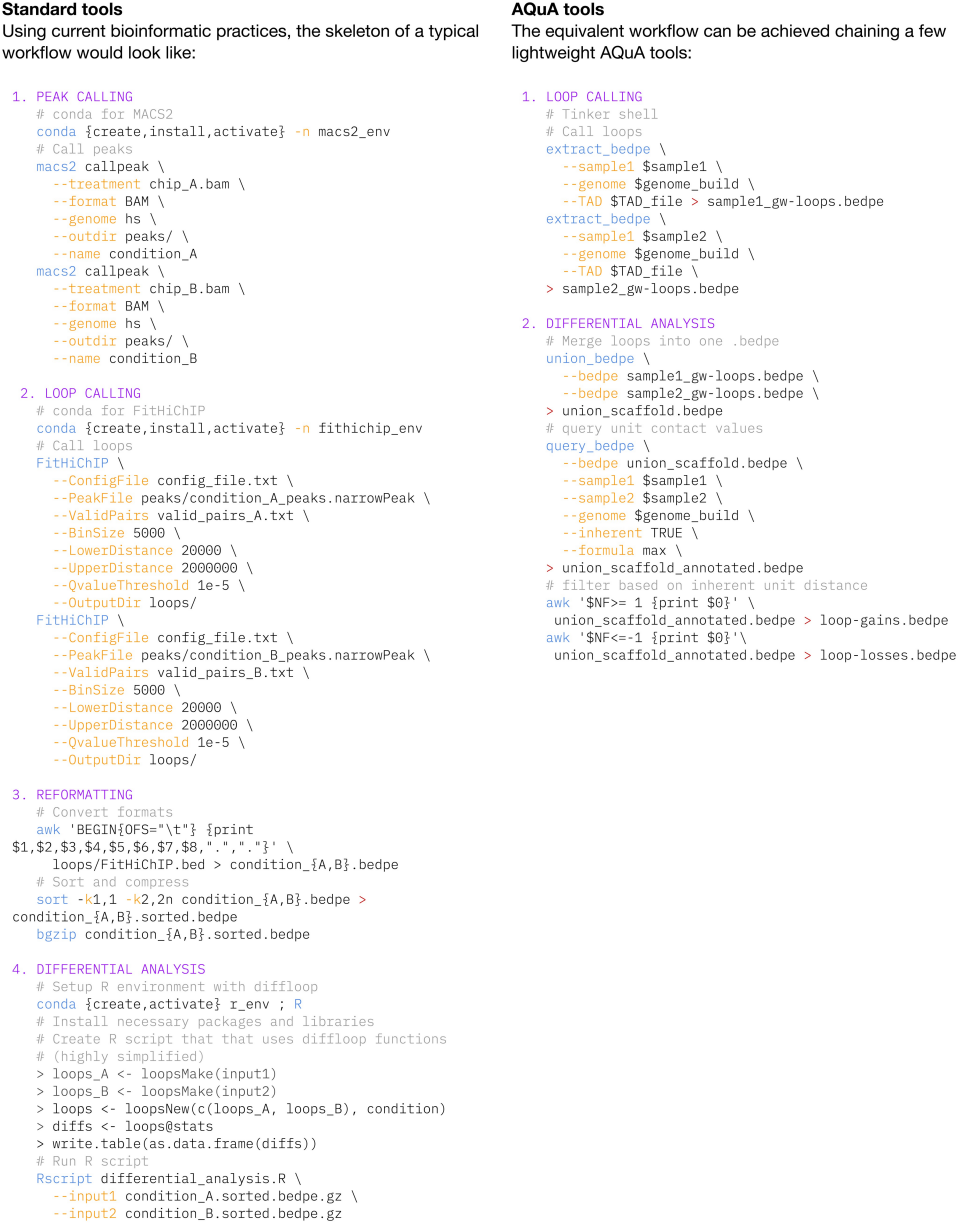
Side-by-side code examples demonstrate the difference in implementation complexity between standard multi-step differential loop calling pipelines and a streamlined AQuA Tools workflow. A standard example workflow (left) using common practice algorithms requires integrating multiple tools across different environments and programming languages, while chaining of select AQuA tools (right) can perform similar analyses in compact lines of code.

One of the standard questions in 3D genomics is differential loop calling between two samples (e.g. treated versus untreated). Conventional workflows require chaining multiple bioinformatic programs across several programming languages that each carry their version and dependency issues. Maintaining the same hardware and software constraints in a container, a differential loop analysis using AQuA tools takes ∼4 min to execute, compared to ∼120 min using standard practices (Fig. 7).

## 4 Normalizations

AQuA tools come with flexible normalization parameters that report back contact values in various numeric spaces. These include “*none*” to work with raw read-count values, “*cpm*” (counts per million) to normalize for sequencing depth, and “*aqua*” for spike-in normalization. Samples without spike-in default to CPM normalization and samples with spike-in default to AQuA normalization.

## 5 Availability and setup

Instructions on how to use AQuA tools can be found at github.com/axiotl/aqua-tools and the tools can be used directly at tinker.axiotl.com on publicly available samples.

## 6 Discussion

The development of AQuA tools has been driven by a desire for clarity and reliability in the tools we use for 3D genomics. We want our tools to be useful in exploratory analyses, directly executed by users, as well as being embedded in complex pipelines. Finally, we want tools that are well documented and easy to access, tools that ease the learning curve of 3D genomics.

Clear and consistent naming of programs and parameters aim to make code written with AQuA tools more readable, reduce the barrier to entry, and facilitate ideation of novel analyses. Reliable algorithms aim to offer product-grade software that can become a solid foundation for other creative 3D genomics tools, with educational materials, documentation and support to ensure continued use and community participation. Bioinformatic packages usually come with little guarantees and are often not actively maintained after publication, generating uncertainty and overhead for bioinformaticians.

Exploratory analysis requires tools that provide results and visualizations within minutes to seconds. AQuA tools are especially responsive when used at the locus level (a few Mb). We intend to create a real-time analysis loop for the user, where ideas can easily be explored on-the-fly and the impact of changing the parameters of an analysis are immediately clear. We named our platform Tinker in order to prime users toward low-stakes, iterative analyses that can lead to a state of flow.

We have set up AQuA tools so that anyone with a browser can run a 3D genomics analysis in a way that obviates the details of compute and storage infrastructure, library management, data sharing, etc., allowing researchers to focus the majority of their bandwidth on the biological questions they aim to answer. The Tinker platform provides all the resources necessary to get started in 3D genomics, including a rich documentation, publicly available HiChIP datasets and a curated set of reference genomic annotations such as TSSs, gene bodies and TADs.

It is difficult to truly understand gene regulation when thinking linearly about the genome. The identification of novel targets for therapeutics relies critically on the correct assignment of pathogenic variants to their driver gene among dozens of other neighboring genes and across hundreds of kilobases. These critical 3D contacts that mediate risk or oncogenicity are highly context-specific, most often distal, sparse in space and sporadic in time. Assigning the wrong gene, e.g. the closest gene, to a risk variant does not just defer the true discovery but might waste precious research resources or derail a PhD or postdoc. We have invested in developing clear and reliable tools for 3D genomics, because we believe that we will only make sense of human health and disease in the light of 3D genomics.

## Supplementary Material

btaf510_Supplementary_Data
