# Supplementary material for: AQuA Tools: clear and reliable BEDPE operations for 3D genomics
Source: Bioinformatics. 2025 Sep 17;41(9):btaf510. doi: 10.1093/bioinformatics/btaf510 (PMC12453685; doi:10.1093/bioinformatics/btaf510)
Supplement: btaf510_Supplementary_Data [file btaf510_supplementary_data.docx]

**Supplementary Material**

# **Inherent Normalization for HiChIP Contact Matrices**

## Background and Motivation

HiChIP experiments yield contact matrices with complex, daisy-chain-like asymmetrical 2D structures that reflect a factor-bound interactome, distinct from the symmetric features observed in HiC data that range from AB compartments, TADs and loops. A critical challenge in HiChIP analysis is distinguishing biologically meaningful chromatin interactions from distance-dependent background noise while enabling meaningful comparison across samples and experimental conditions. While existing HiChIP loop callers successfully model distance-dependent interaction decay through statistical approaches, these methods rely on modeling technical covariates or fitting background distributions to distinguish significant interactions from noise. A fundamental challenge remains in establishing biologically meaningful significance thresholds that can be consistently applied across samples and experimental conditions without requiring extensive parameter tuning.

## Inherent Normalization Framework

The foundation of inherent normalization rests on identifying stable biological references that exist within every epigenomic sample. We exploit the well-established observation that certain genomic elements exhibit predictable activity patterns across cell types: constitutive promoters remain active across virtually all cellular contexts, while cell-type-specific enhancers are predominantly inactive in any given sample.

Using curated sets of DNase I hypersensitive sites (DHSs) and candidate cis-regulatory elements (cCREs) from the ENCODE-3 project, we define reference element sets: constitutive promoters ("on" elements) that are active virtually in all cell types, and cell-type-specific enhancers ("off" elements) that are active in only 2-4 reference samples. The method identifies a biological "ruler" that is inherent within every sample—the distance between the average activities of these "on" and "off" element sets, hence the name of the algorithm. Reads from ChIP-seq of active epigenomes can be poured onto any scaffold (.bed file), and transformed using the sample-specific inherent ruler as follows:

$$inherent score = ( read value - mean(off) ) / ( mean(on) - mean(off) )$$

This transformation places all read values on a 0-1 scale where 0 represents cell-type-specific activity and 1 represents constitutive promoter activity. The approach uses the sample's own biological architecture as the reference standard, making the numeric space and therefore thresholding across a cohort of samples biologically interpretable and universally meaningful **(Fig S1)**.


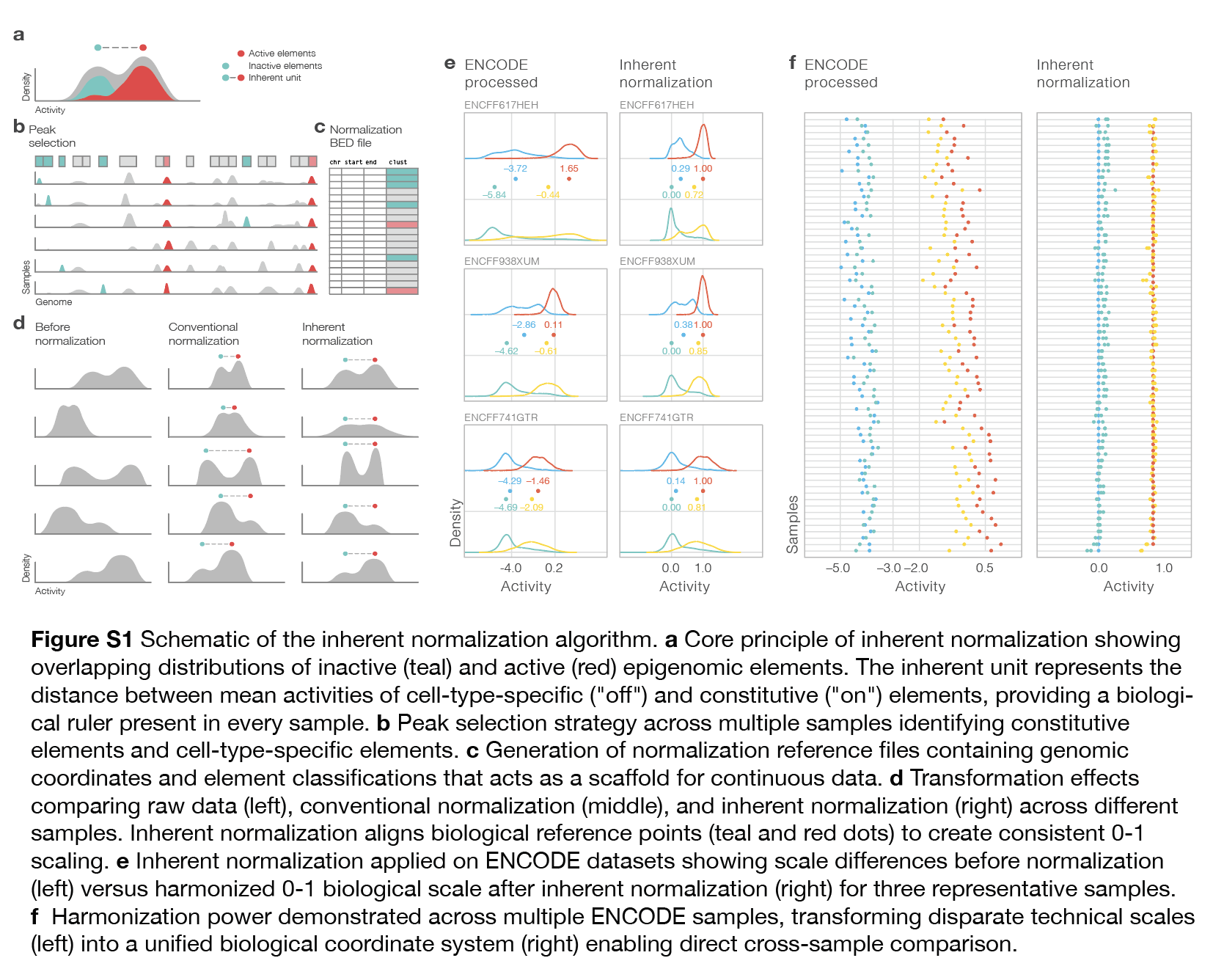


We extend this framework to HiChIP, treating read values on the diagonal of contact matrices as linear, one dimensional epigenomic activity in binned coordinate spaces. Contact values at distances off the diagonal represent epigenomic activity in two dimensions and exhibit the characteristic power-law decay patterns. Therefore, each independent distance index from the diagonal carries different magnitudes of activity, and we calculate on and off pivot values for each distance index independently, allowing us to place any contact value onto the 0-1 scale.

### I. Diagonal Pivot Identification

We first apply inherent normalization to the diagonal bins (1kb or 5kb) of the HiChIP contact matrix, treating each diagonal bin as a 1D epigenomic element. Diagonal bins with inherent scores >0.8 are classified as "on-pivots" (constitutive-like activity), while bins with scores <0.2 are classified as "off-pivots" (cell-type-specific activity). These pivots serve as anchor points representing distinct classes of chromatin environments.

### II. Distance-Specific Power-Law Calculation

For each pivot, we extract interaction frequencies stemming from the pivot at increasing genomic distances (bin_0, bin_1, bin_2, etc.), creating matrices where rows represent individual off-pivots and columns represent distance bins. Off-pivot interactions provide a "floor" estimate of baseline contact frequency—the interaction pattern expected from cell-type-specific regions. On-pivot interactions provide a "ceiling" estimate, but we apply stringent filtering to retain only values ≥2 standard deviations above corresponding off-pivot values, ensuring capture of strong interactions.

The mean across the pivot matrices yields two distance-specific power laws that characterize the sample's interaction landscape:

- **Off-power law:** Background (floor) interaction decay from cell-type-specific regions
- **On-power law:** Enhanced (ceiling) interaction decay from constitutive regions

### III. Distance-Dependent Normalization

For any raw contact value at a binned distance from diagonal d, normalization proceeds as:

$$inherent score_{d} = ( contact value- mean(of{f)}_{d}) / (mean(o{n)}_{d} - mean(of{f)}_{d})$$

transforming any value at distance d. The distance between these curves at each genomic distance becomes the inherent “ruler” for any contact value at that distance, onto which the value can be placed and transformed onto a 0-1 scale **(Fig S2)**. This creates a uniform numeric space that respects natural interaction decay while enabling meaningful comparison across genomic distances and samples.

##
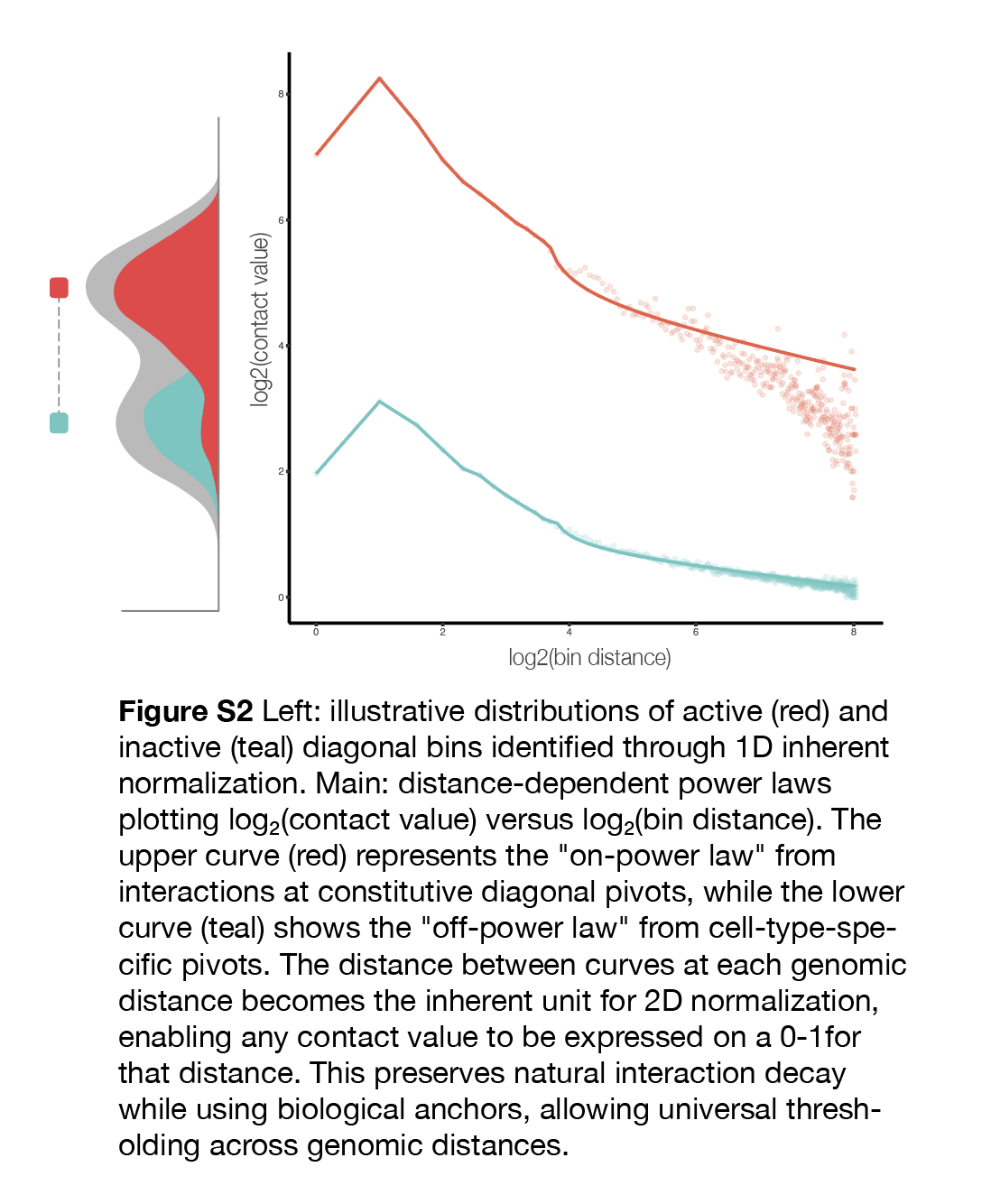


## Implementation in *extract_bedpe*

The *extract_bedpe* tool applies this framework and comes with flexible options to report bin coordinates. Sample-specific inherent power laws are pre-computed and made accessible to the tool so that the 0-1 numeric transformation occurs on-the-fly. Bin coordinates that exceed a user-supplied inherent threshold (default 0.7) at all distances (in a given TAD file or supplied range) are printed in standard-out in .bedpe format **(Fig S3)**. The tool supports multiple output modes to capture different interaction patterns:

- **loop mode:** Individual pixel-level interactions above threshold
- **glob mode:** Agglomerated interactions in any 2D direction, controlled using a radius parameter
- **minimal mode:** Interactions that intersect at long and narrow projections of contact signal

Users can specify genomic ranges or TAD boundaries as the 2D space within which the tool will extract the BEDPE, and a score threshold corresponding to the 0-1 numeric space. For example, a threshold of 0.7 captures interactions that are 70% of the way from baseline to maximally enhanced contact frequency for any genomic distance. The resulting BEDPE output contains interaction coordinates with associated inherent scores, allowing researchers to apply consistent significance criteria across multiple samples.

##
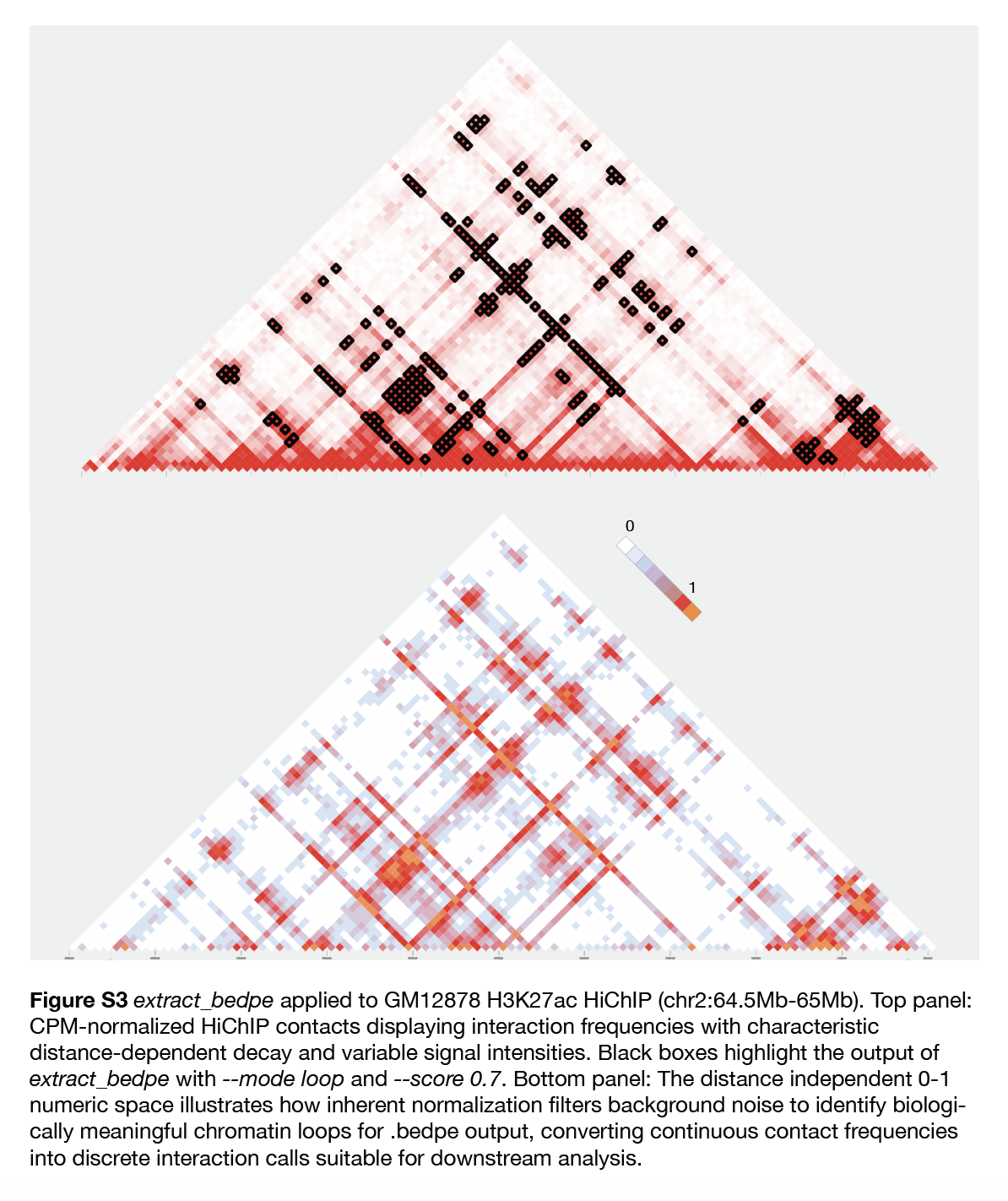


## Advantages

This approach offers several key benefits over conventional normalization strategies. First, it uses sample-internal biological standards, making it robust to experimental variation that carry their own noise. Second, it preserves the natural distance-dependence of chromatin interactions while enabling universal significance thresholds—researchers can use the same inherent score cutoff across all genomic distances. Finally, it produces biologically interpretable scores where values approach 0 for baseline interactions and 1 for maximally enhanced interactions, facilitating intuitive parameter selection and meaningful cross-sample comparison.

The inherent normalization framework implemented in *extract_bedpe* thus enables researchers to extract chromatin interactions from HiChIP data, addressing a fundamental challenge in 3D genomics analysis while providing a foundation for quantitative comparison across experimental conditions and biological contexts.
